# Erie County Med. Society

**Published:** 1852-02

**Authors:** 


*Erie Co. Med. Society.*—The annual meeting of this Society was held on the second Tuesday in January. A well written and able address was read by Dr. Charles Winne, of this city.

The following Officers were elected for the ensuing year:—

*President*—Dr. Ham, of Williamsville.

*Vice President*—Dr. Strong, of Buffalo.

*Secretary*—Dr. Newman, do.

*Treasurer*—Dr. Bailey, do.

*Librarian*—Dr. Samo, do.

*Primary Board*, for the examination of Students entering on the study of Medicine—Drs. Ring, Eastman, and Hawley, of Buffalo.

*Censors*—Dr. Hamilton, of Buffalo, Dr. House, of Springville, Dr. Van Pelt, of Williamsville, Dr. Congar, of Buffalo, Dr. Treat, of do.

